# ECO, the Evidence & Conclusion Ontology: community standard for evidence information

**DOI:** 10.1093/nar/gky1036

**Published:** 2018-11-08

**Authors:** Michelle Giglio, Rebecca Tauber, Suvarna Nadendla, James Munro, Dustin Olley, Shoshannah Ball, Elvira Mitraka, Lynn M Schriml, Pascale Gaudet, Elizabeth T Hobbs, Ivan Erill, Deborah A Siegele, James C Hu, Chris Mungall, Marcus C Chibucos

**Affiliations:** 1 Institute for Genome Sciences, University of Maryland School of Medicine, Baltimore, MD 21201, USA; 2 Swiss Institute of Bioinformatics, 1015 Lausanne, Switzerland; 3 Department of Biological Sciences, University of Maryland Baltimore County, Baltimore, MD 21250, USA; 4 Department of Biology, Texas A&M University, College Station, TX 77840, USA; 5 Department of Biochemistry and Biophysics, Texas A&M University, College Station, TX 77840, USA; 6 Molecular Ecosystems Biology, Lawrence Berkeley National Laboratory, Berkeley, CA 94720, USA

## Abstract

The Evidence and Conclusion Ontology (ECO) contains terms (classes) that describe types of evidence and assertion methods. ECO terms are used in the process of biocuration to capture the evidence that supports biological assertions (e.g. gene product X has function Y as supported by evidence Z). Capture of this information allows tracking of annotation provenance, establishment of quality control measures and query of evidence. ECO contains over 1500 terms and is in use by many leading biological resources including the Gene Ontology, UniProt and several model organism databases. ECO is continually being expanded and revised based on the needs of the biocuration community. The ontology is freely available for download from GitHub (https://github.com/evidenceontology/) or the project’s website (http://evidenceontology.org/). Users can request new terms or changes to existing terms through the project’s GitHub site. ECO is released into the public domain under CC0 1.0 Universal.

## INTRODUCTION

Biocuration is the process whereby information about biological entities is collected and stored. Ideally, this is done electronically, in a standardized format, and kept in a central biological data repository so that it may be easily accessed by the research community (1). Over the past few decades, many such databases have been built to host biomolecular sequence, phenotype, neuroscience, behavioral, medical and other data. The process of biocuration involves making one or more descriptive statements about a biological/biomedical entity, for example that a protein performs a particular function, that a specific position in DNA is methylated, that a particular drug causes an adverse effect or that bat wings and human arms are homologous. Such statements, or assertions, are represented as annotations in a database by representing the entities and assertions using unique identifiers, controlled vocabularies and other systematic tools. Since knowing the scientific basis for an assertion is of vital importance, a fundamental part of the curation process is to document the evidence (2,3). Evidence comes from many sources including, but not limited to, field observations, high-throughput sequencing assays, sequence orthology determination, clinical notes, mutagenesis experiments and enzymatic activity assays. The Evidence and Conclusion Ontology (ECO) provides a controlled vocabulary for the systematic capture of evidence information.

**Figure 1.**
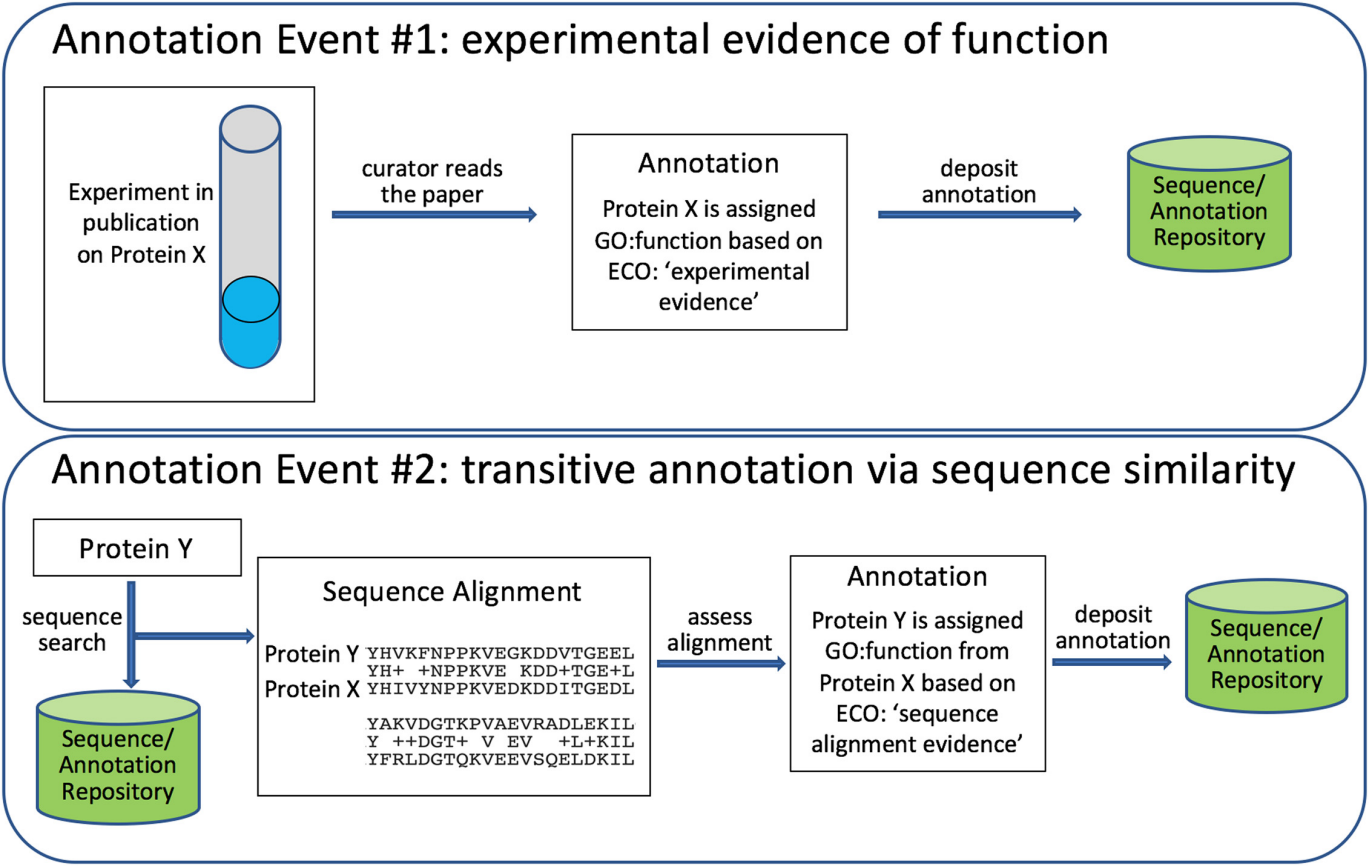
Two annotation examples. In Annotation Event #1, the annotation is made by a curator who has read a published experimental characterization of a protein. In Annotation Event #2, the annotation is made in a transitive manor based on sequence similarity.

An annotation statement consists of at least three elements: the object being annotated, an aspect of the object that is being asserted and the evidence supporting the assertion. The Gene Ontology (GO) (4,5) is in common use for capture of three aspects of gene products: molecular function, biological process and location in cellular component. To illustrate an example annotation (Figure 1), imagine a paper is published that experimentally characterizes the function of Protein X. A curator reads the paper and assigns a GO function term to Protein X based on the experimental evidence in the paper. This information is captured with appropriate GO and ECO terms and deposited in a repository accessible by others. This is an example of biocuration from the literature. Now, further imagine that a curator has primary sequence data for Protein Y, but no publications describing its function. To gain information that might help the curator figure out the function of the protein, they search Protein Y against a database of annotated proteins using a sequence alignment tool. The curator finds that Protein Y has a strong match to Protein X. The curator can then transitively assign the function from Protein X to Protein Y based on their sequence similarity, a type of evidence. Figure 1 illustrates these two annotation examples.

Biological assertions/annotations are routinely used for hypothesis generation as well as multiple kinds of bioinformatic analyses and thus can have far-reaching downstream effects. The ability to fully query and track all aspects of each assertion is important and serves numerous useful purposes. For example, queries of annotation databases allow one to know which types and sources of evidence were used for annotations, either individually or in aggregate. In addition, since many annotations are made in a transitive way (i.e. by propagating the annotation from one biological entity to another biological entity based on some determination of similarity) evidence documentation allows the tracking of provenance of information and chains of annotation. Thus, if one link in that chain is later shown to be invalid (for example, a paper characterizing the function of a protein is found to be in error) it is then possible to quickly find all assertions that were based on that erroneous data and remove or correct them. Further, representing evidence information in a computationally friendly manner allows the establishment and enforcement of curation rules to promote and ensure best practices in annotation. For example, a group could establish that if a function is asserted for a protein based on sequence similarity to another protein, it is required that the matching protein must itself be experimentally characterized. The GO has established such rules (6).

Since evidence comes in many flavors and its consistent storage is of such importance to effective annotation, a systematic way to organize types of evidence and apply them in annotations is essential. An ontology is the best solution to this challenge. An ontology is a controlled vocabulary of terms (or classes) related to each other in defined ways. The ECO (2) provides terms that are used to represent evidence information. ECO has become the community standard for evidence and is used by multiple major data resources such as UniProt (7) and the GO (4,5). Here we provide an update on the status of ECO, our current areas of focus and future plans.

## CURRENT STATUS OF THE ONTOLOGY

**Figure 2.**
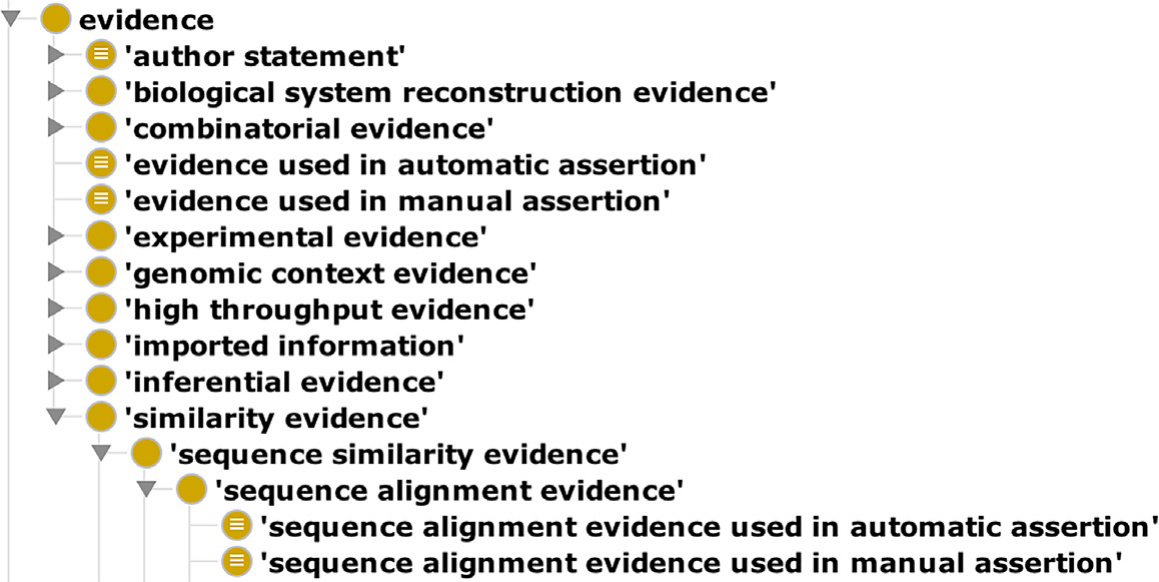
Tree view of ECO terms. This tree view shows all of the first level children of the root node ‘evidence’, showing the variety of types of evidence. In addition, the ‘sequence alignment evidence’ node is expanded showing its parentage back to the root and its two assertion method-specific children.

ECO is structured around two root classes: ‘evidence’ and ‘assertion method’. Terms describing types of evidence are grouped under ‘evidence’ which is defined as ‘a type of information that is used to support an assertion’. All of the first level subclasses of this term can be seen in Figure 2. The second root class, ‘assertion method’, provides a mechanism for recording if a particular assertion was made by a human (i.e. ‘manual assertion’) or if it was done completely in an automated fashion (i.e. ‘automatic assertion’). Whereas, the ‘evidence’ branch of ECO contains dozens of high-level terms with hundreds of children, the ‘assertion method’ branch at present contains just two main subclasses.

Terms from the ‘evidence’ and ‘assertion method’ nodes have been combined to create cross-product terms that describe both features. All leaf nodes in the ontology are either a child of ‘evidence’ and ‘automatic assertion’ or ‘evidence’ and ‘manual assertion’. Although not all types of evidence have yet been used in an automated way, for the sake of logical consistency, all evidence types have been given both a ‘manual assertion’ and an ‘automatic assertion’ child term. Figure 2 shows an example of cross product terms for sequence alignment.

**Figure 3.**
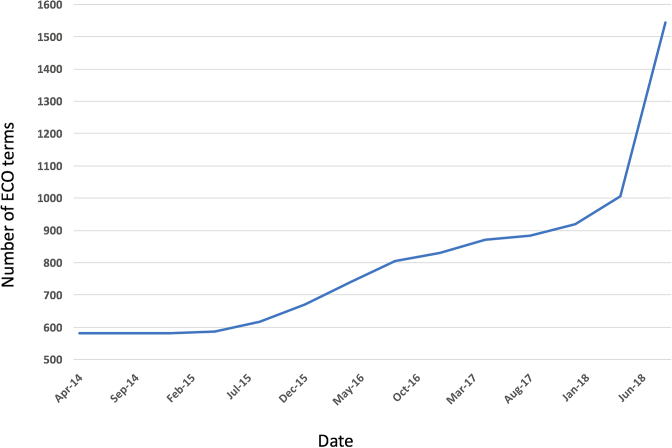
Growth of the ECO. The graph shows total number of terms according to dates taken at 5-month intervals.

Currently, there are 1515 terms in ECO. Since the first ECO publication in 2014 (2), we have increased the number of terms by nearly 1000. Figure 3 describes term growth in ECO since 2014. This term growth has resulted from development efforts in collaboration with several of our user groups (including the Ontology of Microbial Phenotypes (OMP) (8), CollecTF (9), Disease Ontology (10) and GO) as well as from focused development efforts within the ECO team. Some of these efforts will be described in more detail below. In addition, there is a steady flow of requests to our GitHub tracker (https://github.com/evidenceontology/) that results in additional new term generation.

**Figure 4.**
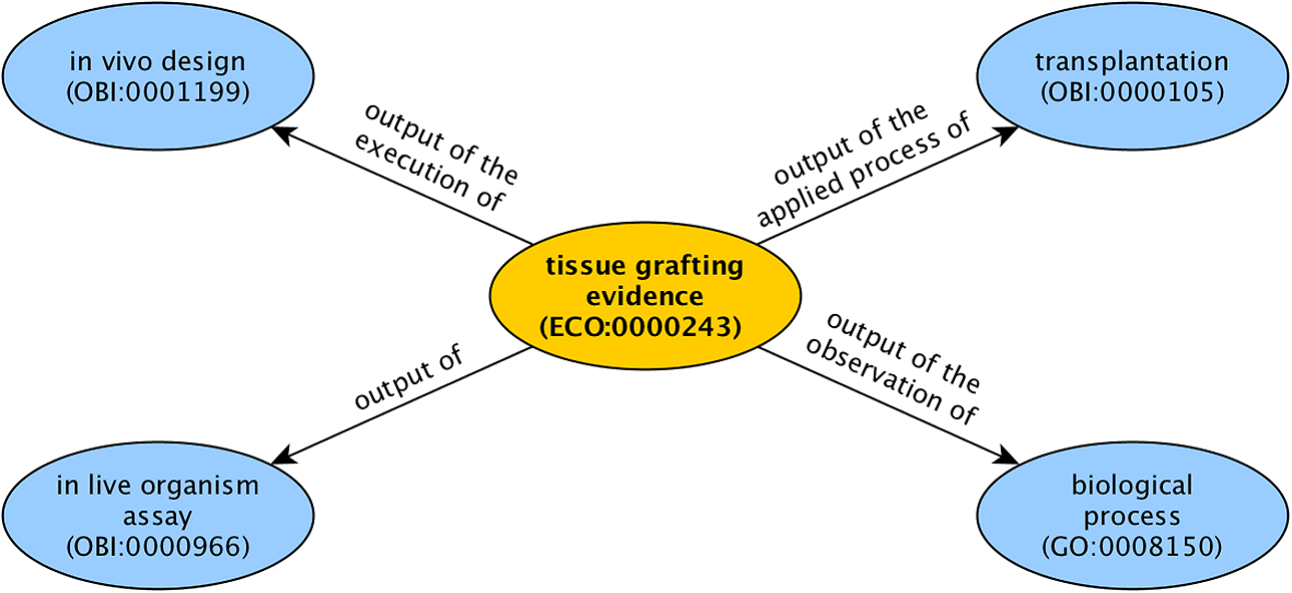
Logical definitions link ECO, OBI and GO terms together.

All 1515 ECO terms have English definitions, most of which are in the Aristotelian (class-subclass) form. Of these, 613 additionally have been assigned logical definitions, i.e. a formal, computable definition distinct from the English one. Of these, 188 have logical definitions that link out to other vocabularies, such as GO and the Ontology for Biomedical Investigations (OBI) (11), and 425 terms have logical definitions that link ECO evidence classes to ECO assertion method classes. An example of one of these logical definitions with linkages to both GO and OBI is found in Figure 4.

## DEVELOPMENT OF THE ONTOLOGY

### Development format and tools

Originally, ECO was developed in the Open Biological and Biomedical Ontologies (OBO) format using the OBO-Edit tool (12). However, neither the OBO format nor the OBO-Edit tool were amenable to achieve the level of expressiveness that is needed for the complex logical definitions and relationships within ECO. Therefore, in 2016 we shifted to the development of ECO directly in the Web Ontology Language (OWL). We use Protégé (13) for viewing the ontology and editing on a small scale. However, the use of Protégé for large-scale additions of terms, or ontology-wide changes, such as those made during the release process, can be tedious and time consuming. Therefore, for larger-scale modifications to ECO we use ROBOT (http://robot.obolibrary.org).

ROBOT is a command line tool developed to support OBO ontologies as part of the OBO Foundry (14). ROBOT contains many useful functions to streamline ontology development and release workflows. For ECO, we make frequent use of the “template" function, which generates terms automatically by converting values in a formatted spreadsheet (the template) into OWL statements. The spreadsheet has defined column headers that are used to specify how the cells are transformed. This is particularly useful when one is developing an entirely new node of the ontology and wishes to add in many terms at one time. Each row of the template represents one new class and the cells of that row contain both logical definitions and class annotations. This function has helped decrease the time associated with adding new terms in bulk. The “template" function can also be used to add new details to existing classes. We have used this feature to add logical definitions based on our collaboration with OBI, described in more detail below.

Another ROBOT function that we employ for our work with ECO is the “extract" function. With our commitment to collaboration and our ongoing work at developing logical definitions that link to other ontologies, we now need to import external ontology classes into ECO. Our main imports at this time are OBI and GO. Both of these, especially GO, contain many classes that are not part of ECO mappings and importing those would unnecessarily increase the size and complexity of ECO. Therefore, we only wish to import a relevant subset of classes using the ROBOT “extract" command. First, we identify the external classes used in ECO and compile these into a simple text file (a line-by-line list of the classes) for each imported ontology. ROBOT then uses these as input to pull the relevant terms and their dependencies from the source ontologies for our use in ECO. ROBOT supports multiple methods of extraction which results in different structures of the import modules. For ECO, we use the MIREOT (Minimum Information to Reference an External Ontology Term) method to extract a simple hierarchy of classes from the import ontology while maintaining all necessary metadata about those classes (http://precedings.nature.com/documents/3574/version/1).

### Increasing logical consistency

During the course of its development, ECO terms have been categorized primarily in one of two ways: the biological context of the evidence (e.g. gene expression or sequence similarity) or the technique or assay used to generate the evidence (e.g. polymerase chain reaction-based evidence). This has created a situation where some terms that have related biological context are found under different unrelated nodes, causing confusion and difficulty for users to find the terms they need. Therefore, we have been screening ECO for grouping terms that describe only a technique or assay and these are being evaluated one-by-one. In general, we find that the children of these grouping terms can be relocated under other more context-dependent parents. At the same time, logical definitions for these terms are created with links to the relevant assay-based OBI terms. The assay-based grouping term is either renamed, merged or made obsolete, depending on the particular case. This ontology review has also led us to find and merge instances of duplicate classes, e.g. ECO:0000295 ‘RNA-seq evidence’ and ECO:0000357 ‘RNA sequencing assay evidence’, which were originally in different parts of the ontology. Changes made as a result of this review will make it easier for users to find the terms they are looking for and will reduce confusion due to ambiguous classes. It also sets a standardized design pattern for adding new evidence terms in the future.

### Infrastructure development collaborations

#### Gene Ontology (GO)

ECO has long supported efforts by the GO Consortium (GOC) to represent evidence in gene product annotations (3,15). Currently, there are over 7 million annotations in the GO repository. As previously reported elsewhere (16), the GOC maintains a column (number six) in its gene association file format, Gene Product Association Data (GPAD) 1.1, that houses ECO identifiers. Here, we provide an update on a previously described (2) collaboratively maintained table that maps the three-letter mnemonic GO evidence codes (e.g. “IDA" or “Inferred from Direct Assay”) to equivalent ECO classes (e.g. ECO:0000314 ‘direct assay evidence used in manual assertion’). This mapping can be found here: http://purl.obolibrary.org/obo/eco/gaf-eco-mapping.txt. Formally linking GO evidence codes to ECO terms facilitates interoperability with other resources that use ECO natively, such as UniProt (7), which also contributed to development of the mapping file. At present, 27 GO evidence codes map to 33 distinct ECO classes by way of either one-to-one mappings or as combinations of GO codes and “GO_references" (citable abstracts describing scientific methods). In all, 46 mappings (i.e. table rows) exist, and more could be added as GO incorporates increasingly diverse evidence types into its knowledge base.

Moreover, ECO maintains an additional file derived from the original mapping table that contains inferred subclass closure: every subclass of an ECO class that is mapped to a GO evidence code also contains a mapping to that same GO code, allowing for efficient bidirectional lookup of evidence (http://purl.obolibrary.org/obo/eco/gaf-eco-mapping-derived.txt). Of the 950 mappings, approximately half (483) map to “Inferred from Electronic Annotation" (IEA, equivalent to ECO:00000501 ‘evidence used in automatic assertion’), which means that the annotation was assigned without a human curator’s judgement. A third of ECO’s classes point to “Inferred from Direct Assay" (IDA) or “Inferred from Experiment" (EXP), with 196 and 110 mappings, respectively. About 5% (56) point to “Inferred from Physical Interaction" (IPI). “Inferred from Expression Pattern" (IEP, 29 mappings) and “Inferred from Mutant Phenotype" (IMP, 23 mappings) together comprise another 6% of the total. The remaining 52 mappings (5%) are scattered amongst the other 21 GO evidence codes.

Annotating evidence with ECO affords more granularity than using GO evidence codes alone. For example, ECO:0007192 ‘motility assay evidence used in manual assertion’ is a cross product term that connotes both that human judgement was involved in making an annotation and that the evidence supporting the annotation arose from some motility assay. This class maps to the GO “Inferred from Direct Assay" evidence code. Thus, annotation with the GO code would indicate that some ‘direct assay’, but not specifically a motility assay, was performed and that human judgement was involved in making the annotation. Hence, a cell motility annotation supported by the ECO term would provide the user with more information about the particular assay involved in generating the evidence than the same annotation supported only by a GO code. Furthermore, although in both cases human intervention is understood to be present, this is more explicitly stated, and made computable, by ECO. In order to share annotations with GO, databases that employ ECO terms use the mapping file to collapse the annotations into the GO system.

GO continues to refine and deepen its evidence annotation, collaborating with ECO to provide the ontological structure. For example, to provide a mechanism to distinguish evidence generated via high-throughput (HT) methodologies from more traditional approaches, several high-level ECO classes were created, starting with ECO:0006055 ‘high throughput evidence’, which is defined as “a type of evidence where data generation is automated with equipment to allow for assaying samples or molecules in parallel". This broadly defined term is parent to subclasses describing HT cell biology evidence, HT mutant phenotype evidence, HT genetic interaction evidence and others. Each HT evidence subtype has a corresponding ‘manual assertion’/‘automatic assertion’ cross-product term, as well. It should be noted that there is active discussion within the biocurator community about the nature of HT data, and this is ongoing work.

#### Ontology for Biomedical Investigations (OBI)

Much recent ECO development has emphasized the normalizing of the ontology in order to facilitate usability and interoperability by broader resources. To accomplish this, ECO has been harmonizing with OBI, an orthogonal ontology in the biomedical domain (11). Whereas ECO covers evidence and assertion methods, OBI provides a mechanism to clearly capture information about scientific investigations through more than 2500 terms describing objectives, assays and devices. While OBI is particularly well-suited to describe instrumentation and research protocols, we have found that users of ECO desire simple representations of evidence that are organized around biological context. Fortunately, complex workflows can be modeled in OBI and linked to ECO terms such that both assay and biological context are captured. We have been collaborating with OBI for several years to create terms in OBI that correspond to assays and protocols underlying terms in ECO. Logical definitions link ECO and OBI terms together (Figure 4). This work has resulted in 188 ECO terms with logical definitions that include OBI terms as well as 41 new terms entered into OBI with 21 more pending. Future work will result in more terms, more linkages between the ontologies and better refinement of both ECO and OBI.

### User-driven term development

As scientists in the community use ECO for their work, they find the need for new terms or for clarification of existing terms and relationships. Requests for new terms and/or term adjustments are regularly posted to our GitHub issue tracker. Addressing these user needs inevitably leads us to discover related areas of the ontology where new terms can be added or improvements made. Thus, our ECO development is dynamic and responsive as the needs of the user community evolve. Below we highlight some of the more large-scale efforts in this area.

#### CollecTF

CollecTF is a resource that focuses on the capture of experimentally characterized bacterial transcription factors, their DNA targets and downstream regulated genes with an emphasis on the experimental provenance of curated records (9). This information is then integrated into central data repositories such as UniProt and the GO Annotation collection (9). Through a detailed curation pipeline, CollecTF biocurators vet and compile published assertions regarding the binding of transcription factors to specific binding sites and their subsequent effects on the transcription of downstream genes. CollecTF initially categorized these assertions using an in-house taxonomy of evidence terms (9). Working in collaboration with the CollecTF team, we have created ECO terms corresponding to the CollecTF controlled vocabulary of experimental techniques (9). This work has resulted in ∼60 terms added to the ontology. CollecTF currently uses these ECO terms in their annotations, enabling the generation of GO annotations from CollecTF records. In further collaboration with the CollecTF group, we have been carrying out manual annotation of published papers specifically to assign ECO terms to sentences. This work has alerted us to numerous areas of the ontology that need further development.

#### Ontology of Microbial Phenotypes (OMP)

The OMP provides terms for the annotation of microbial phenotypes (8). The OMP curators record phenotype annotations by utilizing a wiki-based system. We have created more than 25 new evidence classes in ECO that enabled OMP curators to support annotation assertions. The new evidence classes enriched several parent nodes including ‘immunological assay evidence’, ‘microscopy evidence’ and ‘colony morphology evidence’. Our ongoing collaboration continues to result in new terms that can be used for microbiological annotations.

#### Synapse Gene Ontology and Annotation Initiative

The Synapse Gene Ontology and Annotation Initiative (SynGO) is a collaborative project supported by the GOC, which aims to provide expert curated synaptic gene annotations by utilizing GO (http://www.geneontology.org/page/syngo-synapse-biology). The SynGo uses ECO evidence classes for asserting experimental evidence for synaptic localization and function. We have added more than 10 new evidence classes and revised the classification of some existing classes under ‘direct assay evidence’ and ‘microscopy evidence’ based on the group’s valuable suggestions.

#### neXtProt

neXtProt is a human-centric protein database developed by the CALIPHO group that aims to represent all data relevant to the study of human proteins. For manual curation of protein function and phenotypes, neXtProt has adopted ECO as one of its standardization resources. Recently, neXtA_5_, a semi-automatic annotation application developed through a collaboration between the Swiss Institute of Bioinformatics Text Mining and CALIPHO groups, predicts ECO classes as part of the data extraction step (17,18). We have added nearly 130 new evidence classes to support their curation needs. These classes enhanced many parent nodes including ‘staining evidence’, ‘expression pattern evidence’ and ‘mutant phenotype evidence’.

### ECO releases and availability

As of March 2018, we have fully automated our release process through the use of ROBOT. The release workflow employs multiple ROBOT commands including “report”, “merge”, “reason”, “convert” and “filter” in our Makefile. The Makefile contains a set of “rules" to make “targets" as part of automated build processes. In this case, the rules are various ROBOT commands and the targets are the ECO release files. To generate a release, the developer only needs to run the single command: “make release". As part of our release process, we produce both OBO and OWL formated files through ROBOT “convert". Releases are generally made on a monthly basis and are available in our GitHub repository accompanied by release notes with highlights for that month (https://github.com/evidenceontology). Detailed technical instructions about the ECO release process can also be found on GitHub. The ECO is released into the public domain under CC0 1.0 Universal (https://creativecommons.org/publicdomain/zero/1.0/legalcode). Anyone is free to copy, modify or distribute the work, even for commercial purposes, without asking permission. Ontology files are also available for download from the ECO website (http://evidenceontology.org).

## ECO WEBSITE

**Figure 5.**
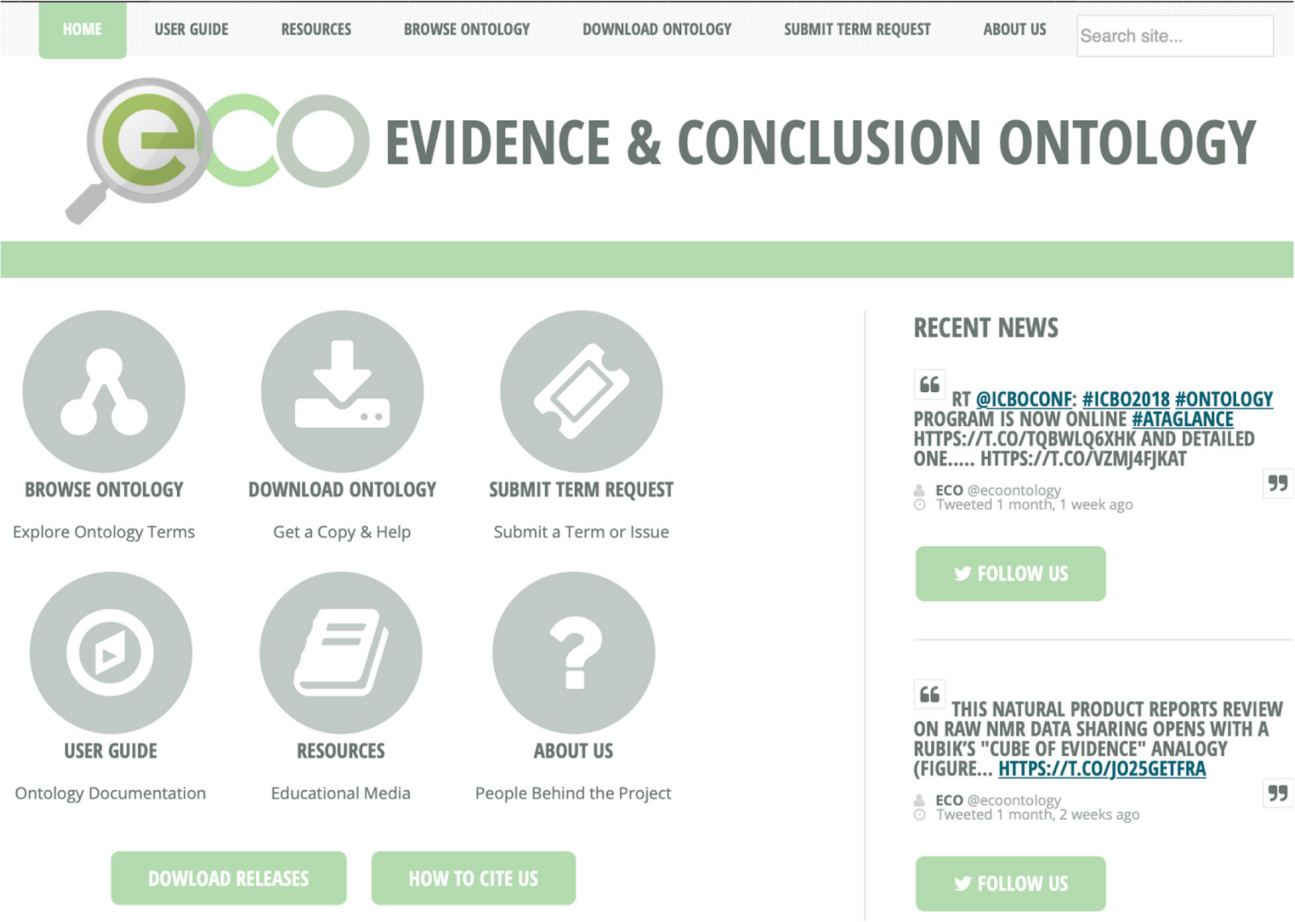
Screen capture of ECO website homepage.

As a resource, ECO aims to maximize its usefulness to users and provide multiple opportunities for engagement. To augment our GitHub development site, which is geared primarily toward developers, we maintain a website (http://www.evidenceontology.org) with additional features for learning more about ECO. Highlights include a user guide with background about ECO and ontologies in the life sciences; links to download the ontologies; instructions on how to submit term requests and feedback; information about ECO staff and users; and a Twitter feed displaying recent tweets by ECO (@ecoontology) (Figure 5).

**Figure 6.**
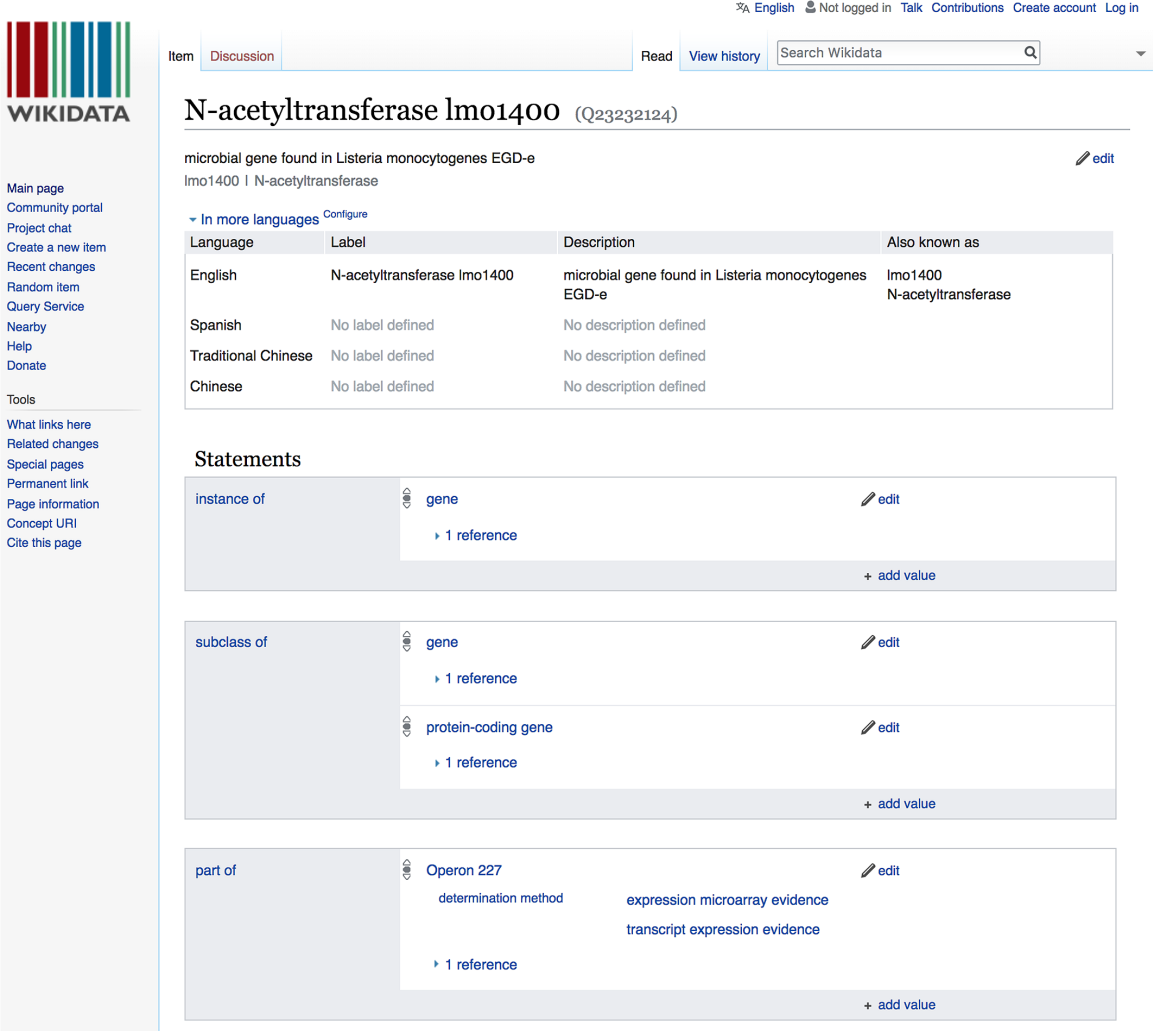
Wikidata annotation for n-acetyltransferase lmo1400.

Prominent on the ECO home page is a link to an ontology browsing tool that employs the Ontology Lookup Service’s (19) GraphView (https://www.npmjs.com/package/ols-graphview) and TreeView tools (https://www.npmjs.com/package/ols-treeview). This makes it very easy for users to quickly find terms of interest for use in annotations or to simply explore ECO. The simple and responsive ontology browser view is helpful for introducing students or interns to the concept of structuring scientific information in addition to give a survey of many types of scientific evidence.

Ontology releases are available at the website in both OBO and OWL format. For users who find that they need a term that is not yet in ECO, there are step-by-step instructions on how to request a term and a link that takes users to our issue tracking system on GitHub where new term requests are submitted and addressed. The GitHub tracker is also where users can request changes to existing terms or post questions or concerns. Additionally, it serves as a forum where the community can discuss the development of ECO.

## USER COMMUNITY

ECO has evolved over the years, not only in terms of the ontology architecture, depth of coverage and external references, but also with respect to the resource’s user community. ECO began as a small in-house ontology, specific to GO annotations made by the model organism databases. Next, it began to be used by gene expression pattern databases (20) and software tools for phenotype analysis (21). This was followed by protein and gene sequence repositories (7) beginning to employ ECO so that they could exchange data openly with GO. In some cases, ECO supplanted in-house evidence vocabularies. As ECO development increased to support these newer users as well as continuing to support GO, a number of small- and large-scale annotation projects and software applications began to use ECO to provide at least part of the computational framework for evidence information. Such projects included many under the auspices of our larger users, for example EBI’s Complex Portal (22) and GO’s Noctua annotation tool (5). To accommodate our ever-increasing group of users, ECO has been iteratively refined and broadened. ECO has sought user feedback the entire time by performing outreach through a number of avenues. Such outreach and development efforts have resulted in greater inclusivity and have brought ECO new users on a consistent basis for the past several years.

In addition to being a production ontology, ECO is also used for teaching as part of an intercollegiate competition focused on genome annotation. Originally piloted by the University of Maryland Baltimore County and the Texas A&M University, the CACAO (Community Assessment of Community Annotation with Ontology)-Phage Hunters competition has now spread to several other colleges (23). This competition pits teams of undergraduate students against each other in a challenge to accurately and completely annotate new phage genomes. CACAO provides a framework for students to submit their annotations to a resource that makes them visible and usable by the scientific community. The annotation process focuses on GO annotations. Students navigate the ECO tree to find terms and their corresponding GO evidence code (which are synonyms attached to the relevant ECO terms) for use in these annotations. This program has turned literature curation and sequence analysis into a team-based challenge that motivates students to become adept at these skills (23).

For a complete list of known ECO users, please visit the ECO website (http://www.evidenceontology.org/about_us/#usergroups). This list was accumulated through literature citations and searching the internet. It is maintained and updated as we learn of new ECO adopters. The list contains references, so that our users may learn how others are using ECO. We encourage anyone using the ontology to contact us via the GitHub tracker (https://github.com/evidenceontology/evidenceontology/issues/new), not only for development requests, but also so that we may know about your project and add it to our list of users.

### Wikidata

A significant step toward broader adoption of ECO by our user community has been its extension beyond the borders of targeted biological databases to a large-scale open data sharing project called Wikidata (24). Wikidata is a central structured data storage repository for all the data from the Wikimedia Foundation (https://www.wikidata.org/wiki/Wikidata:Main_Page). Wikidata is to structured data what Wikipedia is to free text. ECO has become part of the Wikidata ecosystem. Not only has the whole ontology been imported and integrated into Wikidata, but the ECO identifier (https://www.wikidata.org/wiki/Property:P3811) is actively being used to provide machine-readable evidence assertions in Wikidata statements. Each Wikidata statement asserts a particular piece of information about a specific item in a machine-readable manner. ECO has become a part of the Wikidata ecosystem with the aid of the GeneWiki project (25), which is an initiative that strives to create a semantic network of open, accessible and machine-readable information about genes, proteins and diseases.

To illustrate a particular Wikidata annotation, we use the example n-acetyltransferase lmo1400 (https://www.wikidata.org/wiki/Q23232124), which is an instance of a gene and part of Operon 227 (Figure 6). We can also see that this was stated in a peer reviewed research article and, most importantly, what type of determination methods were used to assert this. Such determination methods are all ECO terms. In this case, two determination methods (evidence types) are present: ‘transcript expression evidence’ (ECO:0000009) and ‘expression microarray evidence’ (ECO:0000058). Supporting Wikidata annotations with structured data, including ECO, facilitates provenance tracking for biomedical claims, because it allows users to trace back to the original supporting evidence for a given declaration. Furthermore, any type of biomedical claim that is made can have its determination method traced and be interoperable with all the databases that use biomedical ontologies.

## FUTURE DIRECTIONS

### Continued collaborations with the community

Our work with GO and OBI is ongoing. We also continue to work with many of the users mentioned above and many others at our GitHub development site. We welcome new collaborations and the opportunity to take ECO in new directions. The ECO resource encourages any user faced with documenting scientific evidence in the life sciences to use ECO and contact us through the GitHub tracker.

### Confidence information

Often users view ECO terms as proxies for the level of quality and thus confidence placed on an assertion. However, ECO terms do *not* indicate any level of quality or confidence in the evidence or in the assertion, rather only the type of evidence. One can easily see where the same evidence type (e.g. ‘sequence similarity evidence’) could be of high quality (if the sequences are 95% identical) or of low quality (if the sequences are 20% identical). Therefore another parallel system for capture of confidence information is needed. The Confidence Information Ontology (CIO) (26) proposes one solution to this challenge that involves linking ECO terms and confidence levels as part of the annotation process. We plan to further work with CIO developers to expand the model of how this information will be captured and to pilot its use on real data.

### High-throughput annotations

At the time of this writing, there is ongoing discussion within the biocuration community about the meaning of “high-throughput" and there has been some confusion about the relationship between high-throughput annotations and automatic annotations. Although ECO has terms to describe high-throughput evidence types, we plan to further explore these nodes in collaboration with members of the community who have started to establish guidelines in this area (e.g. the Gene Ontology Consortium).

## DATA AVAILABILITY

ECO is freely available from GitHub (https://github.com/evidenceontology/) and the project’s website (http://evidenceontology.org/). The ECO is released into the public domain under CC0 1.0 Universal.
